# Association of Mediterranean diet adherence with disease progression, quality of life and physical activity, sociodemographic and anthropometric parameters, and serum biomarkers in community-dwelling older adults with multiple sclerosis: a cross-sectional study

**DOI:** 10.1007/s40520-024-02712-y

**Published:** 2024-03-16

**Authors:** Christina Tryfonos, Maria Chrysafi, Sousana K. Papadopoulou, Konstantinos Vadikolias, Maria Spanoudaki, Maria Mentzelou, Dimitrios Fotiou, Eleni Pavlidou, Georgios Gkouvas, Theofanis Vorvolakos, Apostolos Michailidis, Alexia Bisbinas, Olga Alexatou, Constantinos Giaginis

**Affiliations:** 1 Department of Food Science and Nutrition, School of Environment, University of the Aegean, 81400 Lemnos, Myrina Greece; 2 Department of Nutritional Sciences and Dietetics, School of Health Sciences, International Hellenic University, Thessaloniki, Greece; 3 Department of Neurology, School of Medicine, Democritus University of Thrace, Alexandroupolis, Greece; 4 Clinical Dietetics and Nutritional Department, 424 General Military Hospital, Thessaloníki, Greece; 5 Department of Neurology, School of Medicine, Aristoteleio University of Thessaloniki, Thessaloníki, Greece; 6 Department of Geriatric Psychiatry, School of Medicine, Democritus University of Thrace, Alexandroupolis, Greece; 7 1st Department of Pathology, 424 General Military Hospital, Thessaloníki, Greece; 8 University General Hospital of Thessaloniki AHEPA, Thessaloníki, Greece

**Keywords:** Multiple sclerosis, Older adults, Mediterranean diet, Quality of life, Disease progression, Symptoms

## Abstract

**Background:**

Multiple sclerosis (MS) constitutes a chronic inflammatory and degenerative demyelinating disease, which can progressively lead to a broad range of sensorimotor, cognitive, visual, and autonomic function symptoms, independently of patient’ age. However, the clinical studies that examine the role of dietary patterns against disease progression and symptomatology remain extremely scarce, especially concerning Mediterranean diet (MD) in the subgroup age of older adults with MS.

**Aims:**

The present study aimed to investigate the potential impact of MD compliance in disease progression and symptoms severity as well as quality of life and physical activity of community-dwelling older adults with MS.

**Methods:**

This is a cross-sectional conducted on 227 older adults with no history of other severe disease. Relevant questionnaires were applied to collect sociodemographic and anthropometric factors by face-to face interviews between patients and qualified personnel. Serum biomarkers were retrieved by patients’ medical records.

**Results:**

Higher MD compliance was independently associated with younger patients’ age, lower risk of overweight/obesity and abdominal obesity, decreased disease progression and higher muscle mass, as well as greater physical activity, better quality of life, and adequate serum ferritin and albumin levels

**Conclusions:**

MD may exert beneficial effects in older adults with MS. Future strategies and policies are highly recommended to inform both the general population and the older patients with MS for the beneficial effects of MD in preventing MS and in improving or even slowing down the disease progression and symptoms severity of MS.

## Introduction

Multiple sclerosis (MS) constitutes an autoimmune, chronic, inflammatory and degenerative demyelinating disease of the central nervous system (CNS) that can lead to a broad range of sensorimotor, cognitive, visual, and autonomic function symptoms [1]. MS is initially evolved through myelin destruction and subsequent deposits of scar tissue, leading to debilitating physical and cognitive disturbances and a considerable worsening on daily quality of life and performance function [2]. As the MS progresses, symptoms become more prominent, impede the performance of daily activities, and considerably reduce the quality of life [3]. Novel treatment approaches can promote longevity. However, overall mortality rates related with MS have remained almost unaltered over time [4]. Alarmingly enough, the prevalence of MS is constantly increasing, and especially in women, affecting an estimated 2.5 million people worldwide [5].

Disease presentation varies substantially depending on the MS phenotype (i.e., relapsing–remitting [RRMS], primary progressive, secondary progressive, progressive-relapsing, clinically isolated syndrome) in terms of symptoms, pace, and progression [6]. Progressive MS is characterized by consistently worsening disability, which is sufficiently assessed by the Kurtzke Expanded Disability Status Scale (EDSS) [7, 8]. MS seems to be more common in young adults, women, smokers, individuals who have had Epstein-Barr virus, obese individuals, and individuals who live beyond the equator [9, 10].

The most usual established therapeutic approach for MS contains certain immune modulating drugs to lower the relapse risk such as glucocorticoids for the treatment of intense exacerbations, and amantadine for fatigue treatment [10]. Moreover, most treatments aim to maximize recovery from relapses, preventing fatigue and infection, and delaying bedridden disease stages, as no proven treatments currently exist for improving the intense of MS symptomatology [11]. Continuous monitoring with wearable sensors could optimize the management of people with MS, and ocrelizumab, a monoclonal antibody, seems as the most efficient monoclonal antibody for primary PMS, even if it has been associated with higher infection risk [12, 13].

The field of MS has gradually grown considerably during the past 25 years, and especially in the age subgroup of older adults [14]. In fact, the epidemiology of MS has recently shifted to an older population, with a peak prevalence of the disease seen at the age group of 55–65 years [15]. Changes in MS pathophysiology appear to be age dependent and a consistent phase of disability worsening in later stages of life has been identified [16]. Older age has been related with a higher risk of adverse events, including serious infections and MS [17]. Moreover, MS itself may be closely related to cognitive impairment, even though its exact etiopathogenic mechanisms remain still unclear [18]. In addition, during COVID-19 pandemic, hospitalization rate was considerably higher among MS patients, while the pooled-infection rate was estimated to 4% [19].

Furthermore, the older adults constitute a population group with a high prevalence of non-communicable chronic diseases and especially high risk of malnutrition in Europe [20]. Nutritional status also plays a key role on healthy ageing [21]. More to the point, diet quality and quantity are gradually declining in this age group, making the older adults a group at high risk of malnutrition and mortality, worsening health status and quality of life [22]. In this aspect, a cross-sectional study showed that an adequate-nutritional status was associated with better health-related quality of life, higher physical activity, and good sleep quality in older adults [23]. In another cross-sectional study, a high prevalence of malnutrition was recorded in an elderly population sample, which was directly associated with cognitive impairment and depression [24]. Thus, a good-nutritional status should be established by adopting healthy-dietary patterns such as the well-recognized Mediterranean diet (MD), reducing mental health disorders risk [24].

In this aspect, nutrition may act as a possible co-factor, influencing the inflammatory cascade by acting on its molecular pathways and gut microbiota in MS patients [25]. Alarmingly enough, the question whether dietary habits and lifestyle may positively influence the course of MS remains an extremely matter of debate, and MS therapy has currently not been associated with any conclusive and sufficient information on diet and lifestyle [26]. It is, however, frequent that malnutrition may potentially exacerbate MS symptoms, exerting an important role in the development and progression of neurodegenerative diseases, including MS [27].

An appropriate nutritional evaluation of neurodegenerative disease patients and a right nutrition intervention is essential in monitoring their disease. In fact, a high intake of saturated fat increased the incidence of MS, while unsaturated fatty acids may exert a positive effect [28, 29]. In this context, several dietary supplements appear to decrease inflammation and fatigue, also increasing an autoimmunity tolerance in MS patients, and thus improving quality of life and life expectancy, even if the current results remain inconclusive [30]. In addition, only few clinical trials have been performed to address the question of the role of dietary intervention, such as low saturated fat diet in MS treatment, highlighting the strong demand to perform more research to understand the long-term efficacy of dietary interventions in MS patients [31, 32].

Notably, a few clinical studies have demonstrated that a low consumption of saturated fat, low fat vegan, modified Paleolithic, gluten free, MD, and intermittent fasting have been associated with a reduction of MS-related symptoms such as reduced fatigue, improved mood, and improved daily quality of life [33, 34]. However, the above findings have been derived by small pilot studies and did not provide conclusive results. A few recent studies have also assessed the impact of MD on disease progression of MS patients, which cannot establish a causality effect so far [33, 34].

In this aspect, the present cross-sectional study aimed to investigate the potential impact of MD adherence in disease progression and quality of life in an adequate population of 227 MS older adults with no history of any severe diseases except MS. Several sociodemographic, anthropometric, and lifestyle factors were also evaluated by relevant questionnaires, while a series of serum biomarkers were retrieved by MS patients’ medical records..

## Materials and methods

### Study population

#### Study population recruitment

Initially, 395 community-dwelling Caucasian older adults over 65 years old were randomly enrolled from 6 different, geographically diverse Greek regions, both urban and rural, namely Athens, Thessaloniki, Alexandroupoli, Larissa, Patra and Crete. Recruitment to the study was between April 2016 and December 2022 in community-dwelling older adults, being founding mainly during their visits in health care units, as well as in public centers related with entertainment activities for older persons.

**Fig. 1:**
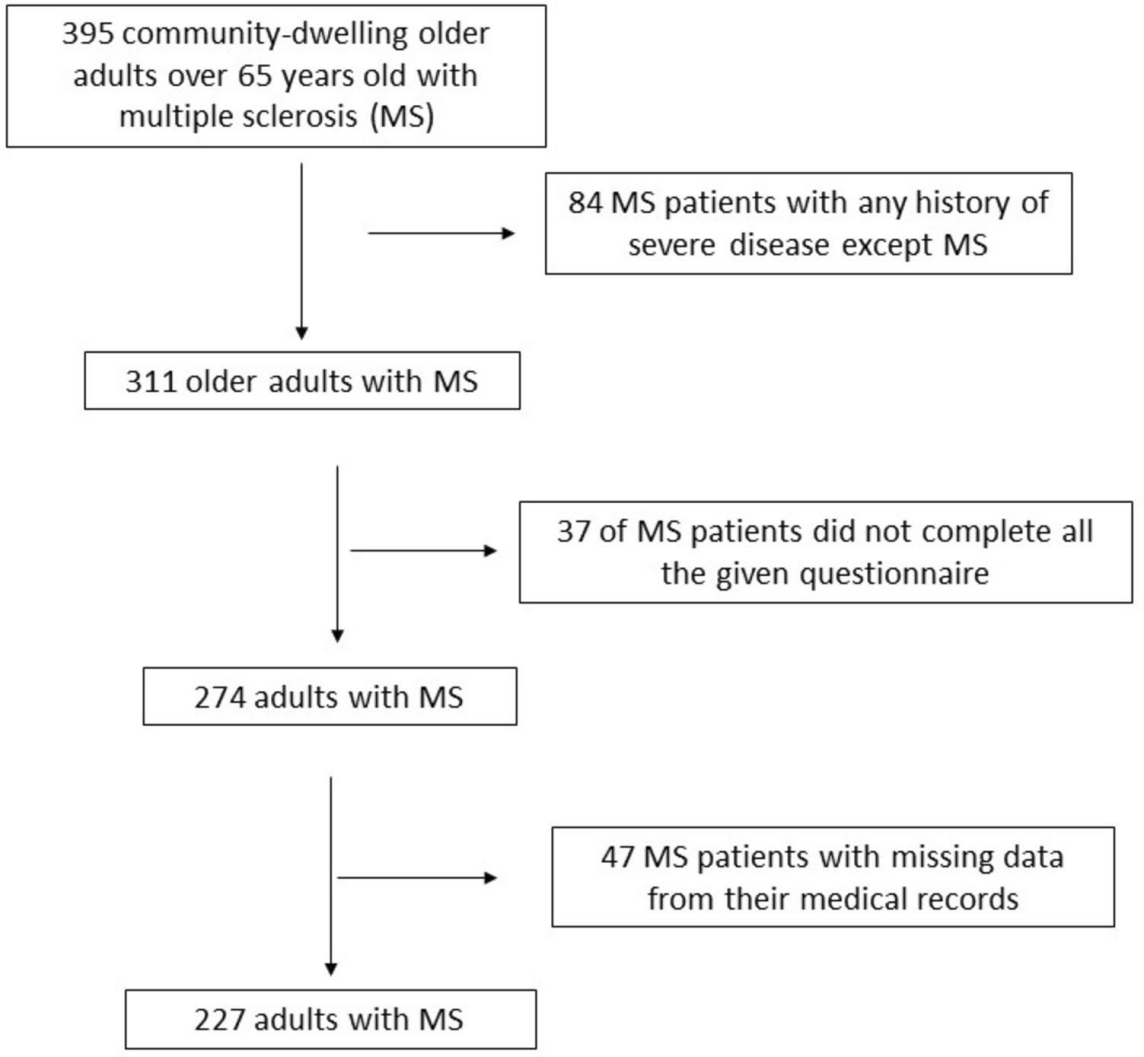
Flow chart diagram of study enrollment

During their thorough recruitment, 84 (21.3%) older adults that had severe, untreated, chronic disease symptoms such as any cardiovascular disease, any cancer or premalignant disease, metabolic disorders, autoimmune diseases, or neurodegenerative diseases were not included in the study. Among the remaining 311 older adults with MS, 37 (11.9%) of MS patients did not complete all the questions of the given questionnaires. Among the remaining 274 older adults with MS, the medical records of 47 (17.2%) patients with MS had several missing data. Finally, 227 older adults diagnosed with MS were included in the final analysis with a final response rate equal to 57.5%. In Fig. 1, a flow chart diagram of the study enrollment is depicted.

#### Ethical approval

All participants’ information was confidential, and all participants were informed about the purpose of the study and signed a written consent for accepting to publish their data. In our study, we carried out all the guidelines of the Declaration of Helsinki in accordance with the World Health Organization (52nd WMA General Assembly, Edinburgh, Scotland, 2000). The Ethical Organization of the University of Aegean (ethics approval code: no 18/22.9.2016) approved the design and the implementation of the present study, as well as the consent approval of the participants.

### Study design

#### Disease disability and symptoms severity assessment

Expanded Disability Status Scale (EDSS) was used for the diagnosis of MS in the enrolled older adults. The EDSS is a sufficient method of quantifying disability in MS and monitoring changes in the level of disability over time [35]. EDSS steps 5.0 to 9.5 are defined by the impairment to walking.

#### Quality of life assessment

The MS Quality of Life-54 (MSQOL-54), a well-recognized, validated questionnaire was used to assess the multidimensional health-related quality of life of the assigned individuals with MS [36]. There is no single overall score for the MSQOL-54. Two summary scores—physical health and mental health—can be derived from a weighted combination of scale scores [37]. In addition, there are 12 subscales: physical function, role limitations-physical, role limitations-emotional, pain, emotional well-being, energy, health perceptions, social function, cognitive function, health distress, overall quality of life, and sexual function [37]. There are also two single-item measures: satisfaction with sexual function and change in health [37].

#### Physical activity assessment

We also assessed physical activity levels utilizing the International Physical Activity Questionnaire (IPAQ) in which subjects mention how much exercise they did in a typical week. This self-administered questionnaire, used worldwide, assesses the overall physical activity over the last 7 days, to categorize it as low, moderate, or high [38]. IPAQ instruments have comprehensively been tested and demonstrated good reliability and acceptable validity properties, at least as good as other self-answered PAQs. Briefly, the purpose of IPAQ-Gr is to sum up vigorous, moderate, and walking PAs over the previous 7-day period and generate a total physical activity score (PAscore), expressed in MET-minutes per week (MET.min.wk^−1^). Based on the IPAQ scoring procedure, PA status is classified into three categories (PAclasses): (1) low PAclass, insufficiently active subjects (total PAscore < 600 MET.min.wk^−1^); (2) moderate PAclass; and (3) high PAclass, HEPA active subjects, (HEPA: health-enhancing physical activity, i.e., total PAscore ≥ 3000 MET.min.wk^−1^ or vigorous PAscore ≥ 1500 MET.min.wk^−1^) [38].

#### Mediterranean diet adherence assessment

For the assessment of the MD adherence, we used the well-recognized Mediterranean Diet Score (MedDietScore) by Panagiotakos et al. [39]. This is a Food Frequency Questionnaire (FFQ) with 11 selected food groups based on Med Diet Score index [39]. In each question were accounted six possible answers, marked from 0 to 5, depending on the level of adherence for each food group. The sum of the 11 questions led to a score from 0 to 55; the higher score represents higher MD adherence. For cereals, potatoes, fruits, vegetables, dairies and olive oil, the scale of six possible responses adjusted per day. For legumes, seafood, red meat and poultry, the scale of six possible answers adjusted per week [39]. The 11th question assessed wine consumption per day with moderate consumption (≤ 1 and ≤ 2 drinks/day for women and men, respectively; one drink = 100 ml = 12 g ethanol) taking the highest score [39].

#### Anthropometric factors assessment

All questionnaires were completed by trained medical personnel (e.g., medical and nursing personnel) and nutritionists and dietitians by face-to-face interviews with community-dwelling older adults to minimize recall bias. The qualified personnel explained in detail to the community-dwelling enrolled older adults all the questions of the questionnaires to ensure the reliability and accuracy of their responses. Body weight and height were also measured at the time of study to calculate Body Mass Index (BMI). Participants weight was determined utilizing a Seca scale [Seca, Hanover, MD], without shoes, to the near 100 g, while height was determined utilizing a portable stadiometer (GIMA Stadiometer 27,335) with no shoes on, to the nearby 0.1 cm. The WHO recommendations were applied to classify the assigned individuals as normal weight, overweight or obese [23, 24, 40]. The waist circumference was measured at the midpoint between the lower margin of the last palpable ribs and the top of the iliac crest, while the hip circumference was measured around the widest portion of the buttocks, with the tape parallel to the floor [23, 24, 40]. The Waist Hip Ratio (WHR) was calculated by dividing waist measurement by hip measurement. In this context, WHR has been found to be superior to BMI [23, 24, 40]. It has been recognized as a greater indicator of abdominal obesity, which is considered as a better anthropometric measure for estimating more efficiently the risk of several cardiometabolic diseases such as diabetes mellitus II [23, 24, 40]. We measured body weight of the participants utilizing the same electronic scale, as well as participants’ height, mid arm, and calf circumference (indicators of muscle mass), utilizing a portable stadiometer (Chapter HM200P, Medi Shop, Greece).

#### Study sample calculation

Sample size calculation was based on the use of PS: Power and Sample Size calculator program, while a simple randomization method was carried with the use of a sequence of random binary numbers (e.g., 010101110 in which 0 represented enrolment and 1 not enrollment to the study). PS software can calculate the sample size required to detect a specified alternative hypothesis with the needed power, the power with which a specific alternative hypothesis can be detected with a given sample size, or the specific alternative hypotheses that can be detected with a given power and sample size.

#### Sociodemographic factors assessment

Patients’ age, gender, educational level, economic status, nationality, living status, and smoking habits were self-reported during face-to-face interviews between participating patients and qualified personnel. Education level was estimated according to the sum of the educational years. Economic status was categorized based on the annual income as: 0 ≤ 5000EUR, 1 ≤ 10,000EUR, 2 ≤ 15,000EUR, 3 ≤ 20,000EUR, 4 ≤ 25,000EUR and 5 ˃ 25,000EUR, according to per capita gross domestic product. We further classified economic status as low for annual income ≤ 10,000EUR, medium for annual income ˃10,000EUR and ≤ 20,000EUR, and high for annual income ˃ 20,000EU.

#### Laboratory parameters

Serum biomarkers levels, including ferritin, albumin, creatinine, Red Blood Count (RBC) Hemoglobin (HMG), hematocrit, Red Cell Distribution Width (RDW), Mean Corpuscular Volume (MCV), Mean Corpuscular Haemoglobin (MCH), Mean Corpuscular Haemoglobin Concentration (MCHC), White Blood Count (WBC), Neutrophils (NEUT), Lymphocytes (LYMPH), Monocytes (MONO), Eosinophils (EO), Basophils (BASO), and Platelets (PLT) were retrieved by the medical records of the enrolled MS patients.

### Statistical analysis

We used Student’s *t* test for continuous variables that followed the normal distribution using Kolmogorov–Smirnov test. We applied Chi-square test for categorical variables. The normally distributed quantitative variables are presented as mean value ± Standard Deviation (SD), and the qualitative variables as absolute or relative frequencies. We performed multiple logistic regression to assess whether MD adherence is independently associated with disease progression, BMI, WHR, muscle mass, quality of life and physical activity after adjustment for multiple confounding factors. As confounding factors, we included only those parameters that showed a significant association with MD adherence in univariate analysis. Multiple regression results are presented as Odds Ratios (OR) and 95% confidence intervals (CI). Differences were considered significant at *p* < 0.05 and 95% Confidence Interval. Statistica 10.0 software, Europe (Informer Technologies, Inc., Hamburg, Germany) was applied for the statistical analysis of the survey data.

## Results

### Descriptives statistics of the study population

The mean age of the enrolled older adults was 71.5 ± 12.6 years old. Concerning patients’ gender, 74.0% were women and the remaining 26% of them were men. The mean educational years of education was 12.5 ± 4.8 years. Regarding economic status, 61.7% of the patients exhibited low annual income, 27.7% of them had a medium annual income and 10.6% of them reported a high annual income. Concerning the living status, 65.2% of the enrolled patients lived with others and 34.8% of them lived alone. As far as smoking habits is concerned, 31.7% of the enrolled patients were regular smokers and 68.3% were never smokers. The vast majority (88.1%) of the assigned patients had a Greek nationality and the remaining 11.9% reported other nationalities.

Among the enrolled older adults with MS, 20.3% were classified as obese, 33.0% were categorized as overweight and 46.7% exhibited a normal weight status based on BMI classification. As far as the abdominal obesity expressed by WHR, 22.0% had high WHR, 40.1% presented moderate WHR and 37.9% exhibited a normal WHR. Concerning the physical activity of assigned older adults with MS according to IPAQ classification, 36.6% of the patients exhibited low-physical activity levels, 33.0% showed medium physical activity levels and 30.4% had high physical activity levels. Regarding, the mid arm and calf circumference (indicators of muscle mass) of the enrolled patients, the 49.3% of them showed < 22 cm mid arm circumference and 46.3% of them had a calf circumference < 31 cm.

As far as EDSS classifications, 43.6% of individuals with MS had a score between 0 and 2.5, 29.1% of them exhibited a score between 3.0 and 4.5, 15.0% presented a score between 5.0 and 6.5 and 12.3% had a score ≥ 7.0. Based on MSQOL-54 classification, 50.7% of enrolled patients with MS had a score below mean value and the remaining 49.3% exhibited a score above mean value.

### Association of MD adherence with sociodemographic and anthropometric characteristics and muscle indices of the study population

In cross-tabulation, higher MD adherence was more frequently observed in younger patients with MS compared to older patients at a significant level (Table 1, *p* = 0.0005). Older adults with MS living with others showed significantly higher levels of MD adherence compared to those living alone (Table 1, *p* = 0.0012). Higher MD compliance was significantly associated with a lower prevalence of overweight and obesity among the older adults with MS (*p* < 0.0001). Abdominal obesity was significantly more frequently observed in MS patients with lower levels of MD adherence (Table 1, *p* < 0.0001). Lower MD compliance was significantly associated with reduced muscle mass as indicated by the mid arm and calf circumference (Table 1, *p* < 0.0001 for both of them). Patients’ gender, educational level, economic status, smoking habits, and nationality did not show any significant association or a trend of correlation with MD compliance (Table 1, *p* ˃ 0.5).

**Table 1:** Association of MD adherence with sociodemographic and anthropometric characteristics, muscle mass indices, disease stage, physical activity, and quality of life levels and serum biomarkers of the study population

Characteristics (*n* = 227)	Mediterranean Diet adherence				
	Very low 54 (23.8%)	Low 56 (24.7%)	Moderate 61 (26.9%)	High 56 (24.7%)	*p*-value^*^
Age (mean ± SD; years)	73.6 ± 12.1	72.5 ± 11.9	70.9 ± 12.5	69.4 ± 12.3	0.0005
Gender (*n*, %)					0.5294
Female	36 (66.7%)	44 (78.6%)	46 (75.4%)	42 (75.0%)	
Male	18 (33.3%)	12 (21.4%)	15 (24.6%)	14 (25%)	
Education level (mean ± SD; years)	12.5 ± 4.4	12.1 ± 5.1	12.8 ± 4.2	12.3 ± 4.6	0.4593
Family economic status (*n*, %)					0.7448
Low	30 (55.6%)	35 (62.5%)	43 (70.5%)	32 (57.1%)	
Medium	17 (31.5%)	15 (26.8%)	13 (21.3%)	18 (32.1%)	
High	7 (12.9%)	6 (10.7%)	5 (8.2%)	6 (10.7%)	
Living status (*n*, %)					0.0012
Living with others	21 (38.9%)	25 (44.7%)	34 (55.7%)	32 (57.1%)	
Living alone	33 (61.1%)	31 (55.3%)	27 (44.3%)	24 (42.9%)	
Smoking habits (*n*, %)					0.2910
No smokers	35 (64.8%)	36 (64.3%)	47 (77.0%)	37 (66.0%)	
Smokers	19 (35.2%	20 (35.7%)	14 (23.0%)	19 (34.0%)	
Nationality (*n*, %)					0.1955
Greek	44 (81.5%)	51 (91.1%)	57 (93.4%)	48 (85.7%)	
Other	10 (18.5%)	5 (8.9%)	4 (6.6%)	8 (14.3%)	
BMI status (*n*, %)					< 0.0001
Normal weight	17 (31.5%)	13 (23.2%)	38 (62.3%)	38 (67.9%)	
Overweight	22 (40.7%)	26 (22.9%)	14 (22.9%)	13 (23.2%)	
Obese	15 (27.8%)	17 (29.9%)	9 (14.8%)	5 (8.9%)	
WHR (*n*, %)					< 0.0001
Low	4 (7.4%)	9 (16.1%)	42 (68.8%)	31 (55.4%)	
Moderate	30 (55.6%)	33 (58.9%)	13 (21.4%)	15 (26.8%)	
High	20 (37.0%)	14 (25.0%)	6 (9.8%)	10 (17.8%)	
Mid arm circumference (*n*, %)					< 0.0001
< 22 cm	35 (64.8%)	36 (64.3%)	21 (34.4%)	20 (35.7%)	
≥ 22 cm	19 (35.2%)	20 (35.7%)	40 (65.6%)	36 (64.3%)	
Calf circumference (*n*, %)					< 0.0001
< 31 cm	31 (64.8%)	33 (64.3%)	19 (40.4%)	22 (31.4%)	
≥ 32 cm	23 (35.2%)	23 (35.7%)	42 (59.6%)	34 (68.6%)	
IPAQ (*n*, %)					0.0027
Low	27 (50.0%)	17 (30.4)	12 (19.7%)	13 (23.2%)	
Medium	15 (27.8%	22 (39.3%)	17 (27.9%)	21 (37.5%)	
High	12 (22.2%)	17 (30.4%)	32 (52.5%)	22 (39.3%)	
EDSS (*n*, %)					< 0.0001
0–2.5	5 (9.3%)	13 (23.2%)	41 (67.2%)	40 (71.4%)	
3.0–4.5	20 (37.0%)	21 (37.5%)	14 (22.9%)	11 (19.6%)	
5.0–6.5	11 (20.4%)	16 (28.6%)	4 (6.6%)	3 (5.4%)	
≥ 7.0	18 (33.3%)	6 (10.7%)	2 (3.3%)	2 (3.6%)	
MSQOL-54 (*n*, %)					< 0.0001
Below mean value	50 (92.6%)	44 (78.6%)	12 (19.7%)	9 (16.1%)	
Over mean value	4 (7.4%)	12 (21.4%)	49 (80.3%)	47 (83.9%)	
Ferritin (mean ± SD; ng/mL)	99.8 ± 53.5	111.0 ± 54.6	129.3 ± 55.3	131.2 ± 53.6	0.0075
Albumin (mean ± SD; g/dl)	3.3 ± 0.7	3.5 ± 0.6	3.6 ± 0.8	3.9 ± 0.5	0.0008
Creatinine (mean ± SD; mg/dl)	0.8 ± 0.6	0.7 ± 0.2	0.8 ± 0.3	0.8 ± 0.5	0.2987
RBC (mean ± SD; 10^6^/mL)	3.9 ± 0.6	4.2 ± 0.5	4.5 ± 0.4	4.7 ± 0.6	0.0012
Hemoglobin (mean ± SD; gr/dL)	13.0 ± 3.4	13.5 ± 4.1	13.7 ± 3.8	14.2 ± 3.3	0.0061
Hematocrit (mean ± SD; %)	35.1 ± 5.1	36.2 ± 5.7	37.9 ± 5.9	38.2 ± 5.4	0.0042
RDW (mean ± SD; fl)	56.5 ± 7.2	57.1 ± 7.4	57.3 ± 7.6	58.1 ± 7.8	0.1031
MCV (mean ± SD; fl)	87.7 ± 8.6	87.2 ± 8.7	86.9 ± 8.5	86.5 ± 8.4	0.3128
MCH (mean ± SD; pg)	28.7 ± 4.3	29.2 ± 4.5	28.9 ± 4.8	29.1 ± 4.3	0.2292
MCHC (mean ± SD; gr/dl)	33.7 ± 4.6	33.5 ± 4.1	32.9 ± 4.3	33.1 ± 4.4	0.3182
WBC (mean ± SD; k/μI)	7.8 ± 2.5	7.7 ± 2.2	7.6 ± 2.6	7.4 ± 2.1	0.0193
NEUT (mean ± SD; %)	67.9% ± 9.1	68.3% ± 9.4	67.9% ± 9.2	67.5% ± 9.8	0.4559
LYMPH (mean ± SD; k/μl)	34.9 ± 4.7	34.1 ± 4.4	34.9 ± 4.6	34.7 ± 4.3	0.3241
MONO (mean ± SD; %)	6.7% ± 1.8	6.5% ± 1.7	6.6% ± 1.9	6.5% ± 1.6	0.5591
EO (mean ± SD; %)	4.0% ± 1.5	3.9% ± 1.7	3.9% ± 1.6	4.1% ± 1.2	0.3965
BASO (mean ± SD; %)	0.6% ± 0.2	0.5% ± 0.3	0.5% ± 0.3	0.6% ± 0.4	0.6903
PLT (mean ± SD; k/μl)	275.4 ± 12.8	274.4 ± 12.3	275.3 ± 12.9	274.9 ± 12.6	0.4231
Vitamin B12 (pg/mL)	548 ± 42.9	562 ± 44.1	574 ± 44.8	582 ± 43.5	0.0345

*BMI* Body Mass Index, *WHR* Waist to hip circumference ratio, *IPAQ* International Physical Activity Questionnaire, *EDSS* Expanded Disability Status Scale, *MS-QLQ27* Multiple Sclerosis-Quality of Life Questionnaire 27 items, *RBC* Red Blood Count, *HMG* Hemoglobin, *RDW* Red Cell Distribution Width, *MCV* Mean Corpuscular Volume, *MCH* Mean Corpuscular Haemoglobin, *MCHC* Mean Corpuscular Haemoglobin Concentration, *WBC* White Blood Count, *NEUT* Neutrophils, *LYMPH* Lymphocytes, *MONO* Monocytes, *EO* Eosinophils, *BASO* Basophils, *PLT* Platelets

^*^Differences were considered significant at *p* < 0.05

### Association of MD adherence with physical activity levels, disease stage and quality of life of the study population

Disease stage of the enrolled patients with MS showed a strong association with their MD adherence (Table 1, *p* < 0.0001). More to the point, patients with MS who higher adopted MD had significantly lower scores concerning EDSS classification (Table 1, *p* < 0.0001). In contrast, patients with a very low or low-MD adherence exhibited significantly higher scores based on ESSD classification (Table 1, *p* < 0.0001). Accordingly, patients adopting MD at higher levels showed significantly greater levels of physical activity compared to those presenting lower MD adherence (Table 1, *p* = 0.0027). Moreover, older adults with MS adopting higher levels of MD showed significantly better quality of life related with MS symptomatology as assessed by MSQOL-54 (Table 1, *p* < 0.0001). In contrast, older adults with MS presenting very low or low-MD compliance exhibited a significantly worse quality of life related with MS symptomatology as assessed by MSQOL-54 (Table 1, *p* < 0.0001).

### Association of MD adherence with serum biomarkers of the study population

Lower levels of serum ferritin, an indicator of iron deficiency, was significantly more frequently observed in patients with reduced MD adherence (Table 1, *p* = 0.0075). Accordingly, lower levels of serum albumin, an indicator of malnutrition, were significantly more frequently noted in patients presenting decreased MD compliance (Table 1, *p* = 0.0008). Reduced levels of serum RBC, hemoglobin, and hematocrit, which are also indicators for iron deficiency, were significantly more frequently observed in patients adopting MD at lower levels (Table 1, *p* = 0.0012, *p* = 0.0061, and *p* = 0.0042, respectively). Higher levels of serum WBC, an indicator of inflammation, were significantly more frequently observed in patients presenting lower MD adherence (Table 1, *p* = 0.0193). Higher levels of serum vitamin B12 were more frequently noted in patients presenting elevated levels of MD compliance (Table 1, *p* = 0.0345). All the other examined serum biomarkers did not show any association or a trend of correlation with MD compliance (Table 1, *p* ˃ 0.5).

### Multivariate analysis of MD adherence by adjustment for multiple confounding factors

In multivariate logistic regression analysis, MD adherence was independently associated with patients’ age, BMI and WHR, disease stage assessed by EDSS, physical activity classified by IPAQ, quality of life of MS patients categorized by MSQOL-54, mid arm, and calf circumference, as indicators of muscle mass, and serum ferritin and albumin levels (Table 2, *p* < 0.05).

**Table 2:** Multivariate analysis of MD adherence by adjustment for potential confounding factors

Characteristics	Mediterranean Diet adherence (Very low + Low vs Moderate + High)	
	OR (95% CI)	*p*-value^*^
Age (over/below mean value)	1.18 (0.89–1.58)	0.0173
Living status (living alone/living with others)	1.58 (1.26–1.89)	0.0132
BMI (overweight + obesity/normal weight)	1.88 (1.62–2.03)	0.0021
WHR (moderate + high/low)	1.75 (1.34–2.16)	0.0149
Mid arm circumference ( < 22 cm / ≥ 22 cm)	1.28 (0.94–1.61)	0.0198
Calf circumference ( < 31 cm / ≥ 32 cm)	1.23 (0.81–1.790	0.0202
IPAQ (low/medium + high)	1.58 (1.23–1.86)	0.0201
EDSS (≥ 5.0 / ≤ 4.5)	2.21 (2.02–2.46)	0.0002
MSQOL-54 (below/over mean value)	2.05 (1.81–2.28)	0.0008
Ferritin (below/over mean value)	1.21 (0.76–1.73)	0.0394
Albumin (below/over mean value)	1.56 (1.29–1.91)	0.0089
RBC (below/over mean value)	1.11 (0.65–1.82)	0.1285
Hemoglobin (below/over mean value)	1.17 (0.71–1.82)	0.2385
Hematocrit (below/over mean value)	1.09 (0.72–1.68)	0.0946
WBC (over/below mean value)	1.02 (0.67–1.71)	0.3869
Vitamin B12 (below/over mean value)	1.22 (0.68–1.94)	0.4837

*BMI* body mass index, *WHR* waist to hip circumference ratio, *IPAQ* International Physical Activity Questionnaire, *EDSS* Expanded Disability Status Scale, *MS-QLQ27* Multiple Sclerosis-Quality of Life Questionnaire 27 items, *RBC* red blood count, *WBC* white blood count, *OR* odds ratio, *CI* confidence interval

^*^Differences were considered significant at *p* < 0.05

More to the point, patients adopting higher MD exhibited a 18% greater probability to be younger (Table 2, *p* = 0.0173). Patients living with others presented a 58% higher incidence of adopting MD at higher levels (Table 2, *p* = 0.0132). Patients with lower MD adherence had an 88% higher risk to be overweight or obese (Table 2, *p* = 0.0021). Accordingly, patients adopting lower levels of MD showed a higher risk of abdominal obesity (Table 2, *p* = 0.0149). Patients presenting lower adherence to MD exhibited at a significant level a twofold higher risk of advanced disease progression (EDSS score (≥ 5.0) compared to those with earlier disease progression (EDSS score ≤ 4.50) (Table 2, *p* = 0.0002). Accordingly, patients with higher MD adherence exhibited a significantly 58% higher incidence of greater physical activity (Table 2, *p* = 0.0201). Moreover, patients with higher adherence to MD showed a twofold higher prevalence of better quality of life compared to those presenting lower levels of MD compliance (Table 2, *p* = 0.0008). In addition, lower MD adherence was significantly associated with a 28% higher prevalence of mid arm circumference < 22 cm (Table 2, 0.0198), and a 23% higher incidence of calf circumference < 31 cm compared to those with higher MD adherence (Table 2, *p* = 0.0202).

Patients adopting MD adherence at higher levers exhibited a 56% greater incidence of higher albumin levels than those presenting lower serum albumin levels (Table 2, *p* = 0.0089). Also, patients with higher MD compliance had a 21% greater prevalence of elevated serum ferritin levels than those with lower serum ferritin levels even if this association was considerably attenuated in multivariate analysis (Table 2, *p* = 0.0394). The other serum biomarkers (RBC, Hemoglobin, Hematocrit, and vitamin B12) did not remain significant in multivariate analysis (Table 2, p˃0.05).

## Discussion

MD is considered as a dietary pattern typical of civilizations localized around the Mediterranean basin, especially Greece, the island of Crete, and southern Italy in the early 1960s [41]. MD includes a high intake of olive oil as the main source of fat, and especially extra virgin olive oil. It also contains high daily intake of plant foods such as vegetables, fruits, legumes, potatoes, bread, and other cereals, nuts, and seeds, as well as fresh seasonal, locally grown, and minimally processed foods [42–44]. In this dietary pattern, dairy products’ consumption is moderate, while seafoods, an excellent source of long-chain polyunsaturated fatty acids, particularly omega-3 fatty acids, and poultry are also consumed in moderate amounts. In contrast, MD includes a low intake of red meat and sweets and moderate consumption of wine at meals [42–44]. Several studies have established the preventing effects of MD against several chronic diseases, such as diabetes mellitus, cardiovascular diseases, metabolic disorders, cancer, mental and psychiatric disorders, aging disorders, and overall mortality [45].

MD has been well-recognized as a dietary pattern that can exert a protective effect on the neurodegenerative process, including MS, due to the fact that it is rich in antioxidants, fiber, and omega-3 polyunsaturated fatty acids [46]. Recent high-quality evidence has currently supported that MD can also promote the reduction of systemic inflammation in several human diseases, including MS [47]. Notably, an increased number of clinical trials in the last decade have provided substantial evidence that the majority of MD beneficial effects could be primarily related to its anti-inflammatory and antioxidant properties as well as to its effectiveness in controlling waist circumference and obesity [48]. These beneficial effects of the MD have mainly been ascribed to its numerous naturally occurring components which exert strong anti-inflammatory and antioxidant properties [49]. Beyond the above effects of MD, this dietary pattern has also been associated with longer telomere length, promoting human lifespan in older adults [50].

In the last decades, there is an increasingly aging global population. However, the way to achieve healthy aging has not still been fully interpreted. The loss of function and frailty syndrome associated with aging increases the vulnerability of the elderly and their propensity to disease [51]. MD has comprehensively been proven to promote healthy aging, increasing the life expectancy of the older population [52]. A substantial-systematic review and meta-analysis has revealed a robust association of a higher adherence to MD with reduced incident of frailty in older adults [53]. Higher MD adherence was also strongly associated with better cognitive status and less-depressive symptomatology in older adults [40]. A recent systematic review and meta-analysis on 31 cohort studies and 5 randomized clinical trials further documented that high adherence to MD was associated with lower risk of mild cognitive impairment and Alzheimer’s disease, promoting better episodic and working memories [54]. Another study has showed that high MD compliance was correlated with favorable quality of life, higher levels of physical activity, and a more adequate sleep quality score in older adults, highlighting its overall benefits in wellbeing in this age group [55]. In addition, a systematic review and meta-analysis have verified that higher MD compliance was associated with improved cognitive function, a lower risk of cognitive impairment as well as decreased risk of dementia, and Alzheimer disease [56]. MD pattern has also been considered as an emerging dietary remedy against sarcopenia of older adults, which further has been established in the present study as we found that higher MD adherence was associated with greater prevalence of adequate muscle mass [57]. Another cross-sectional study conducted on 3450 older adults documented that depression status was independently associated with worse quality of life, poor physical activity, inadequate sleep quality, female gender, overweight and obesity, and living alone after adjustment for multiple potential confounding factors [58]. In accordance with our study, a recent population-based case–control study has also documented that MD was related with less probability of developing MS compared with Western-style diet [59].

In younger MS patients, a recent study conducted on 93 MS patients aged 18–65 years indicated that MD could be an effective nutritional interventional approach in MS patients, which could be related with improved disability level and better quality of life of MS patients [60]. In agreement with our study, another study conducted in a younger MS patients population aged between 18–65 years old and demonstrated that higher MD Adherence Screener (MEDAS) independently predicted better outcomes across MS Functional Composite in 563 consecutive patients. Each MEDAS point was associated with 15.0% lower risk of MS Functional Composite impairment, while higher MEDAS attenuated the impact of progressive disease and longer disease duration on disability [61]. Notably, a multicenter, cross-sectional study conducted on 478 patients with a mean age of 37.99 ± 9.60 years with clinically definite MS has indicated that a low-MD compliance was higher in overweight and obese patients [62]. In a hospital-based, case–control study including 70 MS patients aged from 20 to 60 years, enhanced MD compliance decreased MS risk [63]. Specifically, a consumption of high amounts of fruits and vegetables and higher refined grains consumption were associated, with increased risk of MS [63]. On the other hand, in a multicenter, cross-sectional study, enrolling 427 MS patients with a mean age of 42.41 years, the number of relapses was not affected by MD, while the age of onset of the disease showed a weak correlation with a more delayed time of onset of the disease in patients who consumed foods that are part of the MD [64].

The present cross-sectional study is one of the few studies that assessed the potential-beneficial impact of MD adherence of MS older adults with no history of any severe disease against disease progression, by adjusting for several confounding factors. This study has clearly showed that a higher MD compliance is independently associated with a lower prevalence of advanced disease progression and a lower incidence of related symptoms. Higher MD compliance has also exerted a protecting effect against sarcopenia. In addition, higher MD compliance was independently associated with patients’ age, BMI and WHR, disease stage expressed by EDSS, physical activity classified by IPAQ, quality of life of MS patients categorized by MSQOL-54, and serum ferritin and albumin levels, which are indicators of iron deficiency and malnutrition, respectively. All the above data are in accordance with the previous studies conducted on this field, highlighting MD as a promising nutritional intervention to slow down disease progression of MS and to minimize disease-related symptoms severity. This study also showed that adopting MD may improve the daily quality of life of MS patients.

In this aspect, a recent, comprehensive review highlighted the potential significant role of nutritional lifestyle and physical activity in MS pathogenesis and management [65]. This narrative review underlined that low-carbohydrate, Mediterranean, and fast-mimicking diets have shown both in animal models and in humans a positive effect on MS course and in patient-reported outcomes [65]. The authors supported that MD may be easier to be maintained compared to fast-mimicking and low-carbohydrate diets, which could lead to detrimental side effects, requiring careful-clinical monitoring [65].

The present study has several strengths as it was performed in an adequate sample size of older adults with MS who had no history of sever disease except for MS. This fact has provided substantial evidence for the impact of MD against disease progression independently of comorbidity, which may affect the exact effect of MD against MS [16]. Another strength of our study deals with the assessment of several sociodemographic and anthropometric characteristics and serum biomarkers, which may exert a confounding effect concerning the association of MD with disease progression, symptomatology, and quality of life. Another strength of the study was the use of both BMI and WHR, which reflect both body weight status and the adiposity distribution. A last strength of our study was the used of qualified questionnaires such as EDSS, MSQOL-54, IPAQ and MedDietScore, as well as the performance of face-to-face interviews with the MS patients to increase the reliability and accuracy of their responses. Moreover, the study population was carefully selected to include an equal representation of all age groups beyond 65 years old.

However, the interpretation of the present findings should be made with some limitations in mind. The cross-sectional design of the present study limits the possibility for etiological conclusions and exhibits the potential of recall biases, especially for self-reported questions, even if we performed face-to face interviews. In addition, although BMI is generally considered as a precise and fast indicator for defining body weight of a subject, it cannot assess body composition, which is a more important issue. Thus, bioelectrical impedance analysis (BIA) and/or dual energy x-ray absorptiometry (DXA) should be applied in future studies to enhance the validity of the present results. Furthermore, although a comprehensive approach for confounding adjustment was performed, we recognize the possibility that residual confounding may affect our findings, such as mental health and psychiatric disorders of participants. In this context, psychiatric symptoms are common comorbidities, with depression being the main one [65]. Even though these symptoms are a major determinant of quality of life in MS, they are often overlooked and undertreated [65]. On the other hand, the fact that no conclusions about causality can be obtained due to the cross-sectional design of our study should be emphasized. Drug prescriptions are another potential confounding factor that should be taken into consideration in future studies. Lastly, the association of MD adherence with muscle mass evaluated by mid arm, and calf circumference is only indicative as these parameters are not sufficient to define muscle strength and function.

## Conclusion

This cross-sectional study has provided substantial evidence that adopting MD may slow down the disease progression of MS and may improve the symptoms’ severity in older adults, promoting a better quality of life and increasing physical activity levels. Future well-designed MD intervention studies are highly recommended to evaluate more effectively the potential-beneficial effects of MD in older adults with MS. Overall, an appropriate and balanced diet such as MD could be helpful in improving the condition and well-being of patients with MS, and effectively supporting as a complementary factor to enhance drug therapy efficiency. Prospective studies are very essential to establish a causality effect between MD and MS improvement of disease progression and symptomatology. Future strategies and policies should inform both the general population and the older adults with MS for the beneficial effects of MD in preventing human diseases and in improving or even slowing down the disease progression and the symptoms severity of MS. Moreover, future studies should take into account potential mental disorders and psychiatric symptoms of MS patients, which are common comorbidities, with depression being the main one.

## Data Availability

Study research data are available after request to the corresponding author.
